# High-throughput sequencing offers insight into mechanisms of resource partitioning in cryptic bat species

**DOI:** 10.1002/ece3.49

**Published:** 2011-12

**Authors:** Orly Razgour, Elizabeth L Clare, Matt R K Zeale, Julia Hanmer, Ida Bærholm Schnell, Morten Rasmussen, Thomas P Gilbert, Gareth Jones

**Affiliations:** 1 School of Biological Sciences, University of BristolBristol BS8 1UG, UK; 2 Bat Conservation TrustQuadrant House, 250 Kennington Lane, London SE11 5RD, UK; 3 Centre for GeoGenetics, Natural History Museum of Denmark, University of Copenhagen1350 Copenhagen, Denmark

**Keywords:** Diet, interspecific competition, molecular scatology, next generation sequencing, *Plecotus*

## Abstract

Sympatric cryptic species, characterized by low morphological differentiation, pose a challenge to understanding the role of interspecific competition in structuring ecological communities. We used traditional (morphological) and novel molecular methods of diet analysis to study the diet of two cryptic bat species that are sympatric in southern England (*Plecotus austriacus* and *P. auritus*) (Fig. 1). Using Roche FLX 454 (Roche, Basel, CH) high-throughput sequencing (HTS) and uniquely tagged generic arthropod primers, we identified 142 prey Molecular Operational Taxonomic Units (MOTUs) in the diet of the cryptic bats, 60% of which were assigned to a likely species or genus. The findings from the molecular study supported the results of microscopic analyses in showing that the diets of both species were dominated by lepidopterans. However, HTS provided a sufficiently high resolution of prey identification to determine fine-scale differences in resource use. Although both bat species appeared to have a generalist diet, eared-moths from the family Noctuidae were the main prey consumed. Interspecific niche overlap was greater than expected by chance (*O*_jk_ = 0.72, *P* < 0.001) due to overlap in the consumption of the more common prey species. Yet, habitat associations of nongeneralist prey species found in the diets corresponded to those of their respective bat predator (grasslands for *P. austriacus*, and woodland for *P. auritus*). Overlap in common dietary resource use combined with differential specialist prey habitat associations suggests that habitat partitioning is the primary mechanism of coexistence. The performance of HTS is discussed in relation to previous methods of molecular and morphological diet analysis. By enabling species-level identification of dietary components, the application of DNA sequencing to diet analysis allows a more comprehensive comparison of the diet of sympatric cryptic species, and therefore can be an important tool for determining fine-scale mechanisms of coexistence.

## Introduction

Interspecific competition is an important mechanism structuring ecological communities (Schoener 1983), outweighing the effect of positive mutualistic interactions (Alexandrou et al. 2011), and promoting increased speciation rates (Thierry et al. 2011). Stable coexistence in the face of interspecific competition is the result of ecological niche differentiation, whereby phenotypic character divergence promotes differential resource use, thus reducing the effect of competition for limiting resources (Chesson 2000). There is ample evidence to support both ecological character displacement (species occupying different niches in sympatry; reviewed in Dayan and Simberloff 2005) and limits to the similarity of coexisting competitors (e.g. Kingston et al. 2000). Moreover resource partitioning has been identified as a mechanism that facilitates coexistence in a variety of animal communities (reviewed in Schoener 1974). Nevertheless, some studies have shown that ecologically similar species can coexist through neutral processes that promote phenotypic character convergence rather than divergence (Leibold and McPeek 2006).

Traditional views of niche-based species coexistence are challenged by the presence of sympatric, morphologically similar, but genetically isolated, species (cryptic species) that do not appear to differ sufficiently in their morphology to allow niche differentiation (Wellborn and Cothran 2007). However, morphological similarity may not be a sufficient indication of ecological similarity, and even small differences can influence access to resources (Saunders and Barclay 1992). Cryptic species appear to be common in bats (order Chiroptera) (Clare 2011; Clare et al. 2011a), and in particular among insectivorous bats, because differences in their sensory abilities, in the form of ultrasonic echolocation calls, are not readily distinguished by humans. Moreover, echolocation gives bats an added dimension for niche separation that may be less conserved in its evolution than morphology per se (Jones 1997), and therefore may promote ecological divergence potentially leading to speciation (Kingston and Rossiter 2004). Because foraging habitat use and prey selection in insectivorous bats are closely linked to wing morphology (Aldridge and Rauthenback 1987; Norberg and Rayner 1987) and echolocation call structure (Jones and Rydell 2003), even moderate differences in these features may allow species to partition resources.

Despite their morphological similarity, sympatric cryptic bat species may show pronounced spatial segregation of primary foraging habitats. For example, partitioning of foraging habitat, in the absence of apparent differences in wing morphology (Jones 1997), was identified among the recently separated cryptic *Pipistrellus* species, whereby *P. pygmaeus* selects riparian habitats, while *P. pipistrellus* prefers a wide range of habitats including deciduous woodlands and pasture (Davidson–Watts et al. 2006; Nicholls and Racey 2006). Differences in echolocation call structure can facilitate niche differentiation in sympatric bat species by affecting the detection distances of prey of various sizes (Kingston and Rossiter 2004), as well as contributing to foraging performance under different levels of habitat clutter (Siemers and Schnitzler 2004). For bats foraging in the same habitat, sensory adaptations may allow access to different prey, thus reducing resource competition (Siemers and Swift 2006). However, the role of interspecific competition in driving trophic resource partitioning in bats is still debated (Husar 1976; Arlettaz et al. 1997; Schoeman and Jacobs 2011).

The advent of finer resolution methods of diet analysis may offer a clearer picture of trophic resource partitioning among coexisting species. Indeed some studies that failed to identify resource partitioning among morphologically similar bat species suggest that finer scale differences in microhabitat use or finer resolution diet analysis may reduce the extent of resource overlap (Jacobs and Barclay 2009; Schoeman and Jacobs 2011). Contrary to traditional methods of diet analysis (e.g., fecal or stomach content analysis), polymerase chain reaction (PCR) amplification and DNA sequencing of prey remains from predator fecal samples allows identification to the species level of both soft- and hard-bodied consumed prey (Symondson 2002). Despite the high potential of DNA sequencing to aid identification at finer resolution than the ordinal level for important insect prey, such as Lepidoptera (Whitaker et al. 2009), this technique has only recently been applied to study the diet of insectivorous bats. However, molecular diet studies to date have focused on individual species (Clare et al. 2009, 2011b; Zeale et al. 2011) or on sympatric species that are not close relatives (Bohmann et al. 2011), rather than on cryptic species, where finer resolution of prey types may be fundamental to decipher mechanisms of resource partitioning.

**Figure 1:**
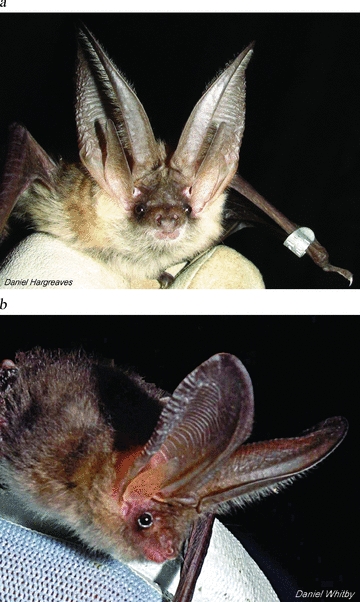
(a) The grey long-eared bat, *Plecotus austriacus*, and (b) the brown long-eared bat, *Plecotus auritus*.

We applied traditional (morphological) and novel next generation sequencing approaches to study the diet of two sympatric cryptic sister species in southern England, the gray and brown long-eared bats, *Plecotus austriacus* and *P. auritus*. *Plecotus austriacus* (Fig. 1) is one of the rarest mammals in Britain, with a prebreeding population estimated at 1000 individuals, and is restricted to southern England, while *P. auritus* is more common and widespread throughout the British Isles (Harris et al. 1995). The long-eared bat genus contains at least 19 distinct cryptic species, nearly all of which were only identified in the past decade based on molecular studies (Spitzenberger et al. 2006; Mayer et al. 2007). Though recognized as separate species in the 1960s (Corbet 1964), *P. austriacus* and *P. auritus* show high overlap in most morphological characteristics (Ashrafi et al. 2010), including their wing morphology (Sevcik 2003) and echolocation call parameters (Russo and Jones 2002). Significant differences in the shape of the baculum (Dietz et al. 2009) may partly account for reproductive isolation between these cryptic species (Patterson and Thaeler 1982). The two bats are sympatric in southern England and parts of Europe. However, while *P. austriacus* is primarily a southern European species, *P. auritus* is abundant in central and northern Europe, but confined to mountainous areas with cooler climates in southern Europe (Spitzenberger et al. 2006). Similarities in morphology and echolocation calls suggest that these species share several niche dimensions, and therefore studying their patterns of resource use may reveal important fine-scale mechanisms of coexistence (Tokeshi 1999).

Our objectives were to assess the ability of PCR-coupled next generation (Roche FLX, Roche, Basel, CH) sequencing to identify fine-scale differences in trophic ecology from fecal samples, and compare the performance of this technique with previous molecular (PCR-coupled cloning and fragment targeting) and nongenetic methods of diet analysis. We hypothesized that despite showing few phenotypic differences, cryptic species are able to coexist due to differences in their ecology. Therefore, we predicted that (1) coexistence among *P. austriacus* and *P. auritus* is mediated via fine-scale mechanisms of resource partitioning; (2) the extent of dietary niche overlap and diet selectivity are affected by variation in prey availability; and (3) the diet of the two bat species corresponds to patterns of foraging habitat selection.

## Methods

### Sample collection

We collected fecal samples directly from 30 *P. austriacus* (representing approximately 3% of the prebreeding British population [Harris et al. 1995]) and 30 *P. auritus* bats caught at sites in southern England (Devon, Isle of Wight, and Somerset). The *P. austriacus* samples were collected between April 2009 and October 2010, and the *P. auritus* samples between June and October 2010 (Supporting Information 1). Bats were caught under license either in roosts, bat boxes, or in woodlands, and held in separate holding bags for a maximum of 30 min. Feces were immediately stored in 100% ethanol in individual tubes, and frozen within 12 h of collection. For the morphological diet analysis, feces were collected once a month between April and October 2009 from seed collection trays placed on the floor of a *P. austriacus* maternity colony in Devon (50°3′N, 3°3′W).

### DNA extraction and PCR amplification

DNA was extracted from feces using the QIAamp DNA stool extraction kit (Qiagen, Valencia, CA) following the modifications described in Zeale et al. (2011). A short (157 bp excluding primer) region of the cytochrome *c* oxidase subunit I (COI) mitochondrial DNA gene was subsequently PCR amplified from each DNA extract. We used generic arthropod primers with a wide taxonomic coverage that includes 13 insect and arachnid orders commonly found in insectivorous bat diets (ZBJ-ArtF1c and ZBJ-ArtR2c; Zeale et al. 2011). The primers were modified into 5′-tagged “fusion primers,” in order to enable Roche FLX sequencing of pools of the amplicons, and subsequent bioinformatic sorting into original PCR (Binladen et al. 2007). Post-PCR amplification, the amplicons were purified, quantified by real-time PCR (qPCR) (Meyer et al. 2007), and then pooled by species at equimolar ratio. Each pool of amplicons was deep sequenced on one-eighth of a Roche GS-FLX platform using Titanium sequencing chemistry. PCR reaction conditions, cycle program, and sequencing procedures followed Bohmann et al. (2011) and are provided in Supporting Information 2.

### Analysis of FLX sequencing data

We used a conservative approach discarding sequences that did not match primers exactly, fragments that were too short, and all singleton sequences (unique sequences that were only present once) to minimize the effect of sequencing errors. Sequences were aligned using the ClustalW algorithm in BioEdit (T. Hall, http://www.mbio.ncsu.edu/bioedit/bioedit.html), and the nucleotide and amino acid alignment was checked by hand with reference to known arthropod sequences. All sequences were collapsed into Molecular Operational Taxonomic Units (MOTUs), approximating species, using the software jMOTU (Jones et al. 2011, https://www.nematodes.org/bioinformatics/jMOTU/). This approach is useful for describing niche breadth and overlap when identification of sequences is limited by an incomplete reference dataset (Clare et al. 2011b). The MOTU assignment was tested at a series of thresholds corresponding to 0–10% sequence divergence and the resulting assignments were graphed. For further analysis, we selected a threshold (2%) based on the infliction point of the graph of distribution of MOTUs recovered, that is, the percent of sequence differences where the graph reached an asymptote and new MOTUs were not being recognized but existing MOTUs had not been collapsed. This value is biologically meaningful and is well within expected variation at the species level in surveyed insects (see Clare et al. 2011b for a discussion of MOTU divergences). Representative sequences from each MOTU (the three most common sequences when identical sequences are collapsed into haplotypes) were compared to reference sequences using the Barcode of Life Data System (BOLD, http://www.barcodinglife.org) identification engine. We used a conservative threshold of >98.5% sequence similarity for species identification based on mean sequence divergence values estimated by Zeale et al. (2011) for the amplified COI region.

Phylogenetic approaches employed by Clare et al. (2009, 2011b) to compliment distance-based species identification were not possible due to the short length of the sequences (157 bp). Moreover, short sequence length meant that some MOTUs gave >98.5% match to more than one species, some of which belonged to different genera or even families. To overcome these obstacles, we used the following criteria to create three identification confidence levels:

1. Solid match (>98.5%) to one species—species-level assignment, or match (>98.5%) to more than one species, all belonging to the same genus—genus-level assignment.
2. Match (>98.5%) to more than one species belonging to different genera, only one of which was a U.K. species—species-level assignment to U.K. species.
3. Match (>98%) to several species of different genera within the same family or to reference sequences only identified to the family-level—family level assignment.

### Diet analysis

The diet of *P. austriacus* and *P. auritus* was quantified based on the percent frequency of occurrence (%FO) of prey orders (the number of feces containing an order divided by the total occurrences of all orders). All other analyses were carried out at the MOTU (species) level.

The extent of dietary specialization and diversity was determined at the MOTU level using the standardized Levins’ measure of niche breadth (Equation 1) and Shannon's diversity index (Equation 2).


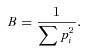
(1)


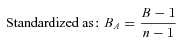
 where *B* is Levins's measure, *p_i_* is the proportion of fecal samples in which MOTU *i* was found, and *n* is the number of possible MOTUs in the diet.


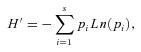
(2) where *p_i_* is the proportion of fecal samples in which MOTU *i* was found.

We used Pianka's (1973) measure of niche overlap (Equation 3) to quantify dietary resource overlap at the MOTU level between the two bat species and within each bat species between the two main collection sites (Devon and Isle of Wight). Null models were used to test whether the extent of niche overlap is greater than expected by chance, and determine the effect of season and sex on dietary resource use. We generate 10,000 simulated matrices of randomized MOTU diet composition, using the software EcoSim (version 7; http://grayentsminger.com/ecosim.htm) with Randomisation Algorithm 3, and compared observed and randomly simulated extents of niche overlap. Bonferroni corrections were applied to retain significance value at *P* < 0.05, resulting in significance level set at *P* < 0.017.


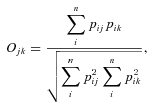
(3) where *P_ij_* is the proportion that resource *i* is of the total resources used by species *j*; *P_ik_* is the proportion that resource *i* is of the total resources used by species *k*; and *n* is the total number of resource states (total number of MOTUs).

Dietary prey composition was compared within each bat species between seasons using nonparametric tests. We compared the proportion of Lepidoptera MOTUs within each fecal sample between the spring (April–May), summer (June–August), and autumn (Sep–Oct) for *P. austriacus*, and between the summer and autumn for *P. auritus*. Statistical analyses were carried out in PASW Statistics 18.

Arthropod prey habitat associations and presence in southern England were determined based on previously published information (Heath and Emmet 1983; Waring et al. 2003; Chinery 2005; Manley 2008; Chandler 2010). Arthropod species commonly found in a range of habitat types were classified as habitat generalists, while species that were exclusively associated with either grassland and open habitats or woodland habitats were classified as grassland and woodland specialists, respectively.

### Morphological diet analysis

#### Primer generality

In order to test for primer amplification biases against certain prey orders, we used traditional microscopic fecal analysis methods (Whitaker et al. 2009) to determine prey composition in all fecal samples analyzed genetically. We looked for the presence of prey orders that were absent from the molecular study and for differences in prey order composition in terms of percent frequency of occurrence (%FO) in diet.

#### Traditional morphological diet analysis

We compared the results of the high-throughput sequencing (HTS) analysis to a previous microscopic fecal analysis study of the diet of *P. auritus* from Somerset, southern England (Hollyfield 1993). However, because there were no previous studies of the diet of *P. austriacus* in Britain, we analyzed feces collected from a maternity colony in Devon, using traditional microscopic fecal analysis methods (Shiel et al. 1997; Whitaker et al. 2009). We randomly selected 30 droppings for analysis from each monthly collection, and teased them apart under a dissection microscope, picking out all recognizable arthropod fragments for identification with reference slides and identification guides (Shiel et al. 1997; M. James pers. comm.). The diets of *P. austriacus* and *P. auritus* were quantified based on percent frequency of occurrence of prey orders (%FO).

## Results

Of the 30 fecal samples collected for each bat species, 28 of the *P. austriacus* (93%) and 24 of the *P. auritus* extractions (80%) produced PCR amplicons, and were therefore included in subsequent analyses. The sequencing run generated 100,508 and 73,606 sequences containing correctly barcoded primers for the *P. austriacus* and *P. auritus* samples, respectively. Subsequent filtering of the data to return only high-quality, identifiable arthropod sequences and removal of singleton sequences reduced these to 91,476 and 63,715, respectively.

The high-quality arthropod sequences were collapsed into 142 MOTUs (using a 2% sequence divergence threshold), belonging to three species of arachnids, two species of Crustacea, and at least six orders of insects. We identified 60% of the MOTUs to the likely species or genus (69% were solid matches—confidence level 1, while 31% matched more than one species, only one of which was a U.K. species—confidence level 2), and 16% to the family level (Table 1). The remaining MOTUs (24%) could not be identified confidently based on our classification scheme and were therefore left as unknown. In Lepidoptera, where the reference dataset is relatively complete, 59 discrete MOTUs were identified as 52 separate species; suggesting that using current taxonomic classification in BOLD, MOTUs slightly overestimated species richness (by 12%).

**Table 1:** List of arthropod prey identified in the feces of 28 *Plecotus austriacus* and 24 *P. auritus* from southern England, including the number of individuals from each bat species that consumed the prey taxa. Confidence levels are based on the BOLD identification system, whereby confidence level 1 = solid match to one species or genus (>98.5%); level 2 = match to more than one species (>98.5%), only one of which was a U.K. species; and level 3 = match > 98% to several species of different genera, or to reference sequences only identified to the family level

Order	Family	Species	*P. austriacus*	*P. auritus*	Confidence level
Lepidoptera	Aractiidae	Unknown	2	0	3
		*Diaphora mendica*	1	0	1
		*Seirarctia* sp.	1	0	1
		*Spilosoma luteum*	3	0	1
	Crambidae	*Agriphila tristella*	2	0	1
		*Chrysoteuchia culmella*	2	0	2
		*Crambus perlella*	1	0	2
	Elachistidae	*Depressaria daucella*	1	0	1
		*Semioscopis* sp.	2	0	1
	Gelechiidae	*Carpatolechia decorella*	1	2	1
	Geometridae	*Gymnoscelis rufifasciata*	1	0	2
	Hepialidae	*Hepialus* sp.	6	2	2
		*Hepialus sylvina*	6	3	1
	Noctuidae	Unknown	6	3	3
		*Acronicta alni*	0	1	1
		*Agrochola litura*	0	1	1
		*Agrochola lota*	0	1	1
		*Agrochola lychnidis*	0	1	2
		*Agrochola macilenta*	0	1	1
		*Agrotis exclamationis*	4	2	1
		*Agrotis ipsilon*	2	0	2
		*Agrotis segetum*	1	0	1
		*Agrotis puta*	1	0	1
		*Allophyes oxyacanthae*	1	2	2
		*Apamea crenata*	0	1	1
		*Apamea epomidion*	0	1	1
		*Apamea monoglypha*	6	0	2
		*Apamea* sp.	1	0	1
		*Autographa gamma*	11	2	1
		*Charanyca trigrammica*	1	0	2
		*Conistra* sp.	0	1	2
		*Diarsia* sp.	0	2	2
		*Hoplodrina ambigua*	1	0	1
		*Hydraecia micacea*	1	0	2
		*Hydraecia* sp.	1	0	1
		*Hypena proboscidalis*	0	1	1
		*Lithophane hepatica*	1	0	2
		*Mythimna albipuncta*	2	0	1
		*Mythimna pallens*	0	1	2
		*Noctua comes*	2	0	2
		*Noctua pronuba*	19	13	1
		*Noctua* sp.	1	1	2
		*Ochropleura* sp.	0	1	2
		*Oligia* sp.	0	1	1
		*Omphaloscelis lunosa*	1	1	1
		*Orthosia gothica*	1	0	2
		*Phlogophora meticulosa*	2	0	1
		*Scoliopteryx libatrix*	1	0	1
		*Tholera cespitis*	0	1	1
		*Tholera decimalis*	2	0	1
		*Xestia xanthographa*	2	6	2
	Oecophoridae	*Diurnea fagella*	0	1	2
		*Endrosis sarcitrella*	0	1	2
Diptera	Anisopodidae	Unknown	0	1	3
	Calliphoridae	Unknown	0	1	3
		*Pollenia* sp.	0	1	2
	Muscidae	Unknown	0	2	3
		*Eudasyphora cyanella*	1	2	1
		*Eudasyphora* sp.	0	1	1
		*Helina impuncta*	1	0	1
		*Morellia simplex*	0	1	1
		*Phaonia subventa*	1	3	1
		*Polietes lardarius*	0	3	1
	Scathophagidae	*Scathophaga stercoraria*	1	1	1
	Syrphidae	*Platycheirus* sp.	0	2	2
	Tachinidae	*Leskia* sp.	1	4	1
	Tipulidae	Unknown	1	0	3
		*Tipula oleracea*	8	1	1
		*Tipula* sp.	1	0	1
Neuroptera	Chrysopidae	Unknown	2	0	3
Coleoptera	Dermestidae	*Anthrenus fuscus*	0	1	1
Hemiptera	Miridae	Unknown	3	0	3
		*Phytocoris tiliae*	1	0	1
Trichoptera	Limnephilidae	*Stenophylax* sp.	1	0	2
Isopoda	Porcellionidae	*Porcellio scaber*	1	1	1
		*Porcellio* sp.	0	1	1
Araneae	Anyphaenidae	Unknown	0	1	3
		*Anyphaena accentuata*	0	1	1
	Tetragnathidae	*Metellina segmentata*	0	1	1

The majority of MOTUs (80%) were consumed by a single individual, though the most common prey species in the diet of both species, *Noctua pronuba*, was consumed by 19 *P. austriacus* and 13 *P. auritus* bats. Number of prey taxa (MOTUs) per dropping ranged between 1 and 17 (means ± SD: *P. austriacus*, 5.3 ± 4; *P. auritus*, 4.2 ± 3).

### Diet composition and specialization

**Figure 2:**
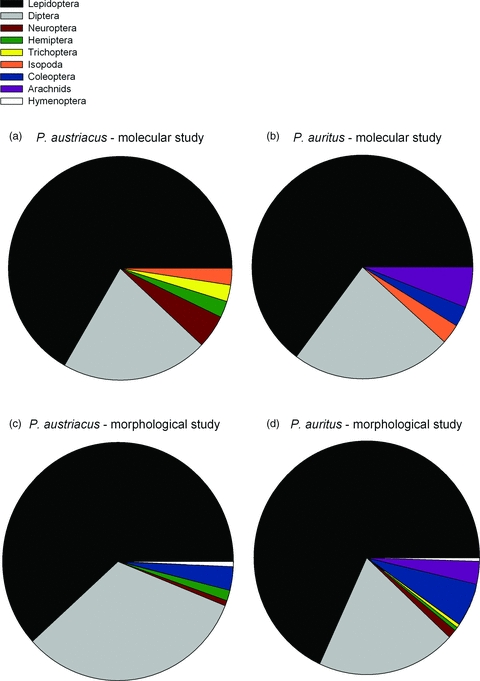
Pie charts showing the diet composition at the ordinal level of the two bat species in southern England based on molecular ([A] *Plecotus austriacus* [*N* = 28]; [B] *P. auritus* [*N* = 24]) and morphological diet analyses ([C] *P. austriacus* from the Devon colony [*N* = 170]; [D] *P. auritus* adapted from Hollyfield 1993 [*N* = 240]). The proportion of prey orders in the diet is presented as percent frequency of occurrence (%FO).

The diet of *P. austriacus* contained 90 MOTUs, while that of *P. auritus* contained 68. Analysis carried out at the MOTU level showed that both bat species had relatively narrow niches (Levins’ measure: *B*_A_ = 0.21, for both species) but high dietary diversity (Shannon diversity index: *P. austriacus H* = 4.1, *P. auritus* H = 3.9). We identified six prey orders in the diet of *P. austriacus* (Lepidoptera, Diptera, Neuroptera, Hemiptera, Trichoptera, and Isopoda) and five orders in the diet of *P. auritus* (Lepidoptera, Diptera, Coleoptera, Araneae, and Isopoda). The diet of both bat species comprised primarily of prey from the orders Lepidoptera and Diptera, with Lepidoptera accounting for 66.7% of the diet of *P. austriacus* and 64.7% of the diet of *P. auritus* (Fig. 2). The same prey orders were identified at similar proportions in the morphological analysis of prey remains in genetically analyzed fecal samples, with the exception of Coleoptera, which was only found in the morphological analysis of the *P. austriacus* samples. However, it is important to note that Coleoptera was only identified in one *P. austriacus* fecal sample, and comprised less than 5% of the volume of that sample.

**Figure 3:**
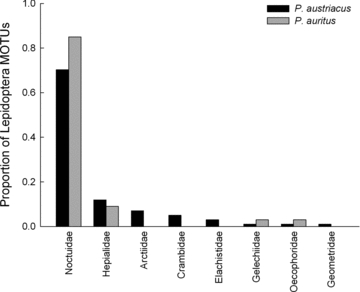
The proportion of Lepidoptera families identified in the diet of *P. austriacus* (black bars, *N* = 28) and *P. auritus* (gray bars, *N* = 24) in southern England.

The majority of the lepidopteran species identified in the diet of both bat species were eared-moths of the family Noctuidae (proportion of Lepidoptera MOTUs: *P. ausitracus* 71%, *P. auritus* 83.3%; Fig. 3). Species of the family Tipulidae were the most common dipterans identified in the diet of *P. austriacus* (76%), while species of the dipteran family Muscidae were more common in the diet of *P. auritus* (65%). The most common prey species in the diet of *P. austriacus* were *N. pronuba*, *Autographa gamma*, *Hepialus* sp., *Apamea monoglypha*, and *Tipula oleracea*, while the most common prey species in the diet of *P. auritus* were *N. pronuba*, *Xestia xanthographa*, *Hepialus sylvina*, and *Polietes lardarius* (Table 1).

**Figure 4:**
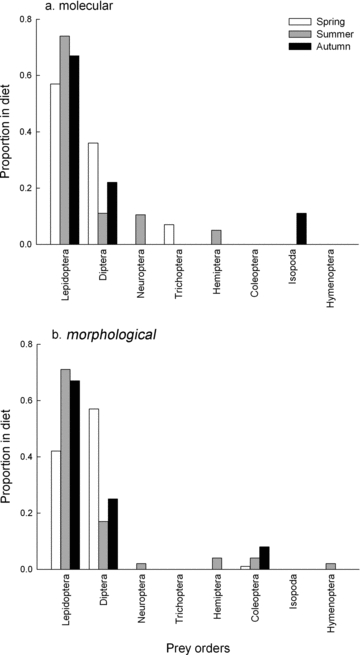
Seasonal variation in the proportion of prey orders identified in the diet of *P. austriacus* in southern England based on (A) the molecular study (Spring: *N* = 8, Summer: *N* = 14, Autumn: *N* = 6) and (B) the morphological study (Spring: *N* = 60, Summer: *N* = 80, Autumn: *N* = 30). Diet composition is presented as percent frequency of occurrence (%FO).

The diet composition at the ordinal level of both bat species differed between the seasons. *Plecotus austriacus* consumed higher proportion of Lepidoptera in summer than in spring (Kruskal–Wallis test: *H* = 7.72, *N* = 14, 8, *P* = 0.002; Fig. 4), while *P. auritus* consumed more Lepidoptera in summer than in autumn (Mann–Whitney test: *U* = 33, *N* = 10, 14, *P* = 0.014; Supporting Information 3).

### Dietary niche overlap

**Figure 5:**
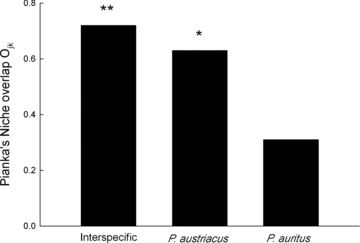
Extent of between (interspecific) and within species niche overlap among *P. austriacus* and *P. auritus* in southern England based on Pianka's measure of niche overlap, including the significance of niche overlap relative to random simulations (**P < 0.001; *P < 0.01). Within species niche overlap was tested by comparing the diet of Devon and Isle of Wight colonies separately for each bat species (*P. austriacus*: Devon *N* = 19, Isle of Wight *N* = 9; *P. auritus*: *N* = 10, 7).

We found little evidence for dietary resource partitioning among these cryptic bat species. Interspecific niche overlap, measured based on MOTUs, was significantly higher than expected by chance (*O*_jk_ = 0.72, *P* < 0.001) and higher than intraspecific niche overlap (*P. austriacus*: *O*_jk_ = 0.63, *P* = 0.009; *P. auritus*: *O*_jk_ = 0.31, not significant) (Fig. 5). Niche overlap between bat species was greater in the summer (*O*_jk_ = 0.62, *P* = 0.012) than the autumn (*O*_jk_ = 0.48, not significant).

### Prey habitat associations

The majority of the prey MOTUs identified to the species level were habitat generalists. Generalist prey species tended to be consumed by both bat species and formed the majority of their diet (*P. austriacus* 65%, *P. auritus* 68%). Of the 25 prey MOTUs with specific habitat associations, the majority of species that the literature considered as found exclusively in woodlands (67%) were consumed by *P. auritus*, while the majority of species associated with more open grassland habitats (75%) were consumed by *P. austriacus*. Patterns of prey selection by habitat were not random (Chi square: χ^2^ = 13.1, df = 1, *P* < 0.01).

### Concordance between morphological and molecular analysis

A morphological analysis of 170 *P. austriacus* fecal samples resulted in the identification of six prey orders, the most common of which was Lepidoptera (62%), followed by Diptera (32%). The morphological analysis identified two prey orders that were not recorded in the molecular diet analysis, Coleoptera and Hymenoptera, but instead did not identify any Trichoptera or Isopoda (Fig. 2). Nevertheless, differences in the proportion of prey orders were not statistically significant (Paired *t*-tests, Arcsin transformation: *P* = 0.98). Similar to the results of the molecular diet analysis, the proportion of Lepidoptera in the diet differed between the study seasons, being significantly lower in spring (Kruskal–Wallis test with Arcsin transformation: *H* = 47.7, df = 2, *P* < 0.001), while the proportion of Diptera was highest in the spring (Fig. 4).

A comparison with a previous morphological study of the diet of *P. auritus* (data adapted from Hollyfield 1993, *N* = 240) showed similar proportions of Lepidoptera (68%) and Diptera (20%) but higher proportions of Coleoptera (6%) than those identified in our molecular study (Fig. 2). The morphological analysis revealed four additional prey orders absent from our study, Neuroptera, Trichoptera, Hymenoptera, and Plecoptera, though all contributed together to less than 1.6% of the diet. As in the diet of *P. austriacus*, there were no significant differences in the results of the molecular and microscopic diet analysis (*P* = 0.59). Niche overlap between the diet of *P. austriacus* and *P. auritus* at the ordinal level, based on the result of the morphological diet studies, was very high and significant (*O*_jk_ = 0.95, *P* = 0.01).

## Discussion

### Use of high-throughput DNA sequencing to study interspecific interactions

High-throughput DNA sequencing technology has the potential to increase the scope of dietary studies by revealing patterns of resource partitioning among competing species with a level of detail not previously possible (Valentini et al. 2009a). This study is the first application of molecular techniques to study interspecific competition between sympatric cryptic species that are expected to show high dietary overlap due to strong morphological similarities (Findley and Black 1983). Unlike previous DNA sequencing techniques, which relied on either cloning (e.g., Deagle et al. 2005; Zeale et al. 2011) or the selection of prey fragments (Clare et al. 2009, 2011b), high-throughput DNA sequencing exploits entire samples and does not rely on subsampling, and therefore even very rare dietary items can potentially be detected (Deagle et al. 2009). Moreover, the use of uniquely tagged primers (Binladen et al. 2007) makes HTS more economical by enabling the pooling of many samples (up to 30 fecal samples per sequencing lane in our study) together while retaining the ability to ascribe prey sequences to an individual bat consumer, thus allowing the identification of seasonal and sexual dietary and niche overlap patterns. Yet, due to differences in primer binding, prey digestibility, and amount of DNA in prey tissue, neither method of molecular diet analysis to date can provide an accurate quantitative measure of diet composition within any one sample (Sipos et al. 2007; Deagle et al. 2010).

The number of potential prey items (MOTUs) identified in the diet of the two bat species in our study (*P. austriacus* [90], *P. auritus* [68]) did not exceed those identified in previous molecular studies of the diet of insectivorous bats (Clare et al. 2009—127 prey species in the diet of *Lasiurus borealis*; Zeale 2011—89 prey species in *Barbastella barbastellus*). However, the number of MOTUs per fecal sample in our study was much higher (we identified a maximum of 17 prey MOTUs per dropping in *P. austriacus* and 16 in *P. auritus*, versus a maximum of seven and nine prey species in Clare et al. (2009) and Zeale (2011), respectively), suggesting that HTS has greater potential to reveal rare dietary components and species with less visible remains in the feces.

The application of molecular techniques did not result in the identification of more lepidopteran prey species than previous studies of culled prey remains. Bauerova (1982) recorded 140 species of Lepidoptera in the diet of *P. austriacus* in central Europe, while Robinson (1990) found 34 species of Lepidoptera in the diet of *P. auritus* in England. However, because DNA sequencing techniques are less likely to be biased toward large hard-bodied prey (Whitaker et al. 2009), we were able to successfully record a variety of smaller and softer bodied prey items of orders overlooked in previous studies.

Strong overall agreement between the diet composition and seasonal patterns of diet composition of the two bat species identified in our HTS study and our own and previous (Hollyfield [1993] for *P. auritus*) morphological diet studies supports the validity of the molecular diet composition estimations at the ordinal level. Moreover, the fact that similar results were obtained from morphological studies carried out on >170 fecal samples suggests that our sample size was sufficient for representing differences in the diets of the two bat species.

The short sequence length (157 bp) and lack of complete overlap between the coverage of the primers used in our study and available COI reference sequences in BOLD (only ∼130-bp overlap) meant that not all MOTUs could be identified to the species level, and we were not able to use phylogenetic trees to confirm identification. In addition, many of the sequences showed 100% match to more than one species distributed globally, some of which belonged to different genera, thus forcing us to create a three-tiered confidence system and to identify some species based on whether they are found in the United Kingdom. Nevertheless, because a high proportion of prey DNA fragments recovered from feces are degraded and relatively short (Deagle et al. 2006), short amplicons may provide a more accurate estimate of diet composition, including also prey of higher digestibility, and are more likely to identify rare dietary components. As such, short amplicons can overcome problems encountered by Clare et al. (2011b) of low amplification success and high contamination by nonprey DNA. The source material may thus dictate the choice of primer length, a trade-off between length of amplicon for identification and the impact of DNA degradation.

As suggested by Zeale et al. (2011), the primers successfully amplified DNA from a wide range of arthropod orders, including a crustacean order (Isopoda) never before recorded in the diets of the two bat species. Zeale et al. (2011) fed an individual *P. auritus* an experimentally manipulated diet of Diptera, Coleoptera, and Lepidoptera, and found close agreement between items recorded in the diet by molecular analysis and the food items fed to the bat 6–24 h previously, suggesting that the primers do not appear to suffer from significant amplification biases. Microscopic analysis confirmed the diet composition results concluded from genetic data. Although previous morphological studies found higher proportions of Coleoptera in the diet of both bat species, our microscopic analysis of genetically analyzed fecal samples confirmed the low proportion of Coleoptera in our samples and the absence of other prey orders. Hence, the primers appear to provide good coverage of the main prey orders commonly found in the diet of insectivorous bats.

Molecular approaches were able to overcome many of the biases associated with traditional bat diet analysis techniques (reviewed in Whitaker et al. 2009), while providing an accurate estimate of diet composition and more fine-scale extent of niche overlap (see Table 2 for comparison of the performance of the various diet analysis methods). Yet, molecular diet analysis is still constrained by an incomplete reference sequences database, though this problem was at least partly overcome in our study through the use of MOTUs as a surrogate for prey species identification. High correspondence between MOTUs and lepidopteran species, for which the BOLD reference dataset is relatively complete, suggests that MOTUs are an effective method of diet assessment. However, our results suggest that they may overestimate prey species richness by 12% due to the proportion of repeated species identification in our study. This overestimation can be attributed to intraspecific polymorphism in the amplified region (Valentini et al. 2009b), though it can also be an artifact of the incomplete reference dataset or taxonomic ambiguity among some prey species.

**Table 2:** Comparison of the strength and weaknesses of available diet analysis techniques

		Molecular techniques		Traditional methods	
			
	High-throughput sequencing (HTS)	Cloning (Zeale et al. 2011)	Fragments (Clare et al. 2011b)	Morphological—feces/stomach	Culled prey remains
Diet resolution	High: species level constrained by reference sequences database (can be mediated by MOTU)	High: species level constrained by reference sequences database (can be mediated by MOTU)	High: species level constrained by reference sequences database (can be mediated by MOTU)	Low: family order	High: species order but depends on taxa
Diet coverage	Better representation of the DNA extracted, but bias from primer binding biases, and relative DNA abundance of prey	Limited by the selected number of clones. Bias toward free floating DNA, DNA abundance, and primer bias	Selected fragments. Bias toward prey of low digestibility or fragment selection method	Greater bias toward hard-bodied prey of low digestibility	Bias toward large prey that requires culling
Diet quantification	Number of species/ MOTUs, no within samples quantification	Number of species/ MOTUs, no within samples quantification	Number of species/ MOTUs, no within samples quantification	Number of prey taxa and percent volume	Number of prey taxa and density
Rare dietary components	High potential to identify rare prey—high proportion of DNA variance sequenced	Selection of clones for sequencing reduces the chance of identifying rare prey	Potential of identification depends on number of fragments sequenced from each dropping (costs)	High potential to identify hard-bodied rare prey, but low potential to recover rare soft prey	Rare prey identified only if require culling
Accuracy	Does not require taxonomists to obtain accurate results, lower analyzer bias	Does not require taxonomists to obtain accurate results, lower analyzer bias	Does not require taxonomists to obtain accurate results, lower analyzer bias	Accuracy requires significant entomological training on the part of the identifier	Accuracy requires entomological training on the part of the identifier
Costs	Less limited by the amount of prey sequences or droppings per sequencing run. Lower cost per sequence but high initial costs and needs specialized facilities	Expensive cloning process, but less specialized facilities	Depends on number of fragments sequenced—each fragment requires separate sequencing. Less specialized facilities.	Very low—minimal consumables	Very low—minimal consumables
Applicability across feeding groups	High relevance across taxa, including herbivores	High relevance across taxa, including herbivores	Mainly relevant for predators and seed disperser	Mainly relevant for predators	Only relevant for predators that use feeding perches

### Trophic ecology relative to resource availability

Insectivorous bats are sometimes perceived as opportunistic foragers (Kunz 1974), consuming prey based on their availability both in the environment (Swift et al. 1985) and to the bat itself, given the constraints of its sensory and flight abilities (Fenton 1990; Siemers and Schnitzler 2004). Clare et al. (2011b) shows that the same predator can be regarded as a generalist, based on the number of prey MOTUs identified in the diet, and as a specialist when the diet is considered phylogenetically, if all the prey species identified in the diet belong to the same order. In our study, diet selectivity at the ordinal, and even family level, appears to be high. Lepidoptera, and in particular eared-moths of the family Noctuidae, were the main prey consumed by both bats. In contrast, when the diet is considered at the MOTU or species level, *P. austriacus* and *P. auritus* appear to have a generalist diet, including a variety of prey items, most of which were often only consumed by a single individual (although if more samples could be obtained more individuals may have consumed these items). Diet generality is reinforced by the fact that all prey MOTUs identified to the species level were common and widespread in southern England, and were consumed by the bats when at the peak of their adult-form activity (Heath and Emmet 1983; Waring et al. 2003).

The larger size and potentially high energetic value of Lepidoptera makes them an important component of the diet of many insectivorous bat species (e.g., Vaughan 1997; Bogdanowicz et al. 1999). Based on a small sample size, we found a trend in the diet of *P. austriacus* and *P. auritus* to shift from a diet dominated by Lepidoptera in summer toward the consumption of high proportions of Diptera during spring and autumn, when Lepidoptera availability is reduced (Jones 1990). This seasonal trend of dietary shift highlights the preferential consumption of Lepidoptera and the tendency of *P. austriacus* and *P. auritus* to concentrate on high-quality prey at times of high energetic demands (Kurta et al. 1989; McLean and Speakman 1999, for *P. auritus*).

Many moths have tympanal organs that enable them to detect the echolocation calls of approaching bats and initiate behavioral responses to evade predation. They are particularly sensitive to relatively high-intensity echolocation calls between 20 and 60 kHz (Rydell et al. 1995; Miller and Surlykke 2001). Consequently, the proportion of Lepidoptera is higher in the diet of bats that either produce echolocation calls above or below these frequencies (Bogdanowicz et al. 1999), or produce low-intensity echolocation calls when approaching prey (Goerlitz et al. 2010), or listen to prey-generated sounds to detect prey (Faure and Barclay 1992). High proportions of Lepidoptera in the diets of *P. austriacus* and *P. auritus* have been attributed to the latter two strategies (Rydell et al. 1995) due to the bats’ low-intensity echolocation calls, long ears, and the ability of *P. auritus* to glean prey from surfaces without producing echolocation calls (Coles et al. 1989; Anderson and Racey 1991). However, *P. austriacus* appears to hunt mainly by aerial hawking rather than gleaning (Bauerova 1982) and the use of passive listening by this species is yet to be confirmed (Swift 1998).

### Mechanisms of coexistence in cryptic bat species

Although dietary partitioning is regarded as a principal mechanism for resource partitioning in animal communities (e.g., Schoener 1974; Saunders and Barclay 1992, for bats), it does not appear to play an important role in facilitating coexistence among sympatric *P. austriacus* and *P. auritus*. High similarity in both morphology (Ashrafi et al. 2010) and echolocation call characteristics (Russo and Jones 2002) suggests that the two species may not differ sufficiently in their aerodynamic or sensory abilities to have access to different prey (Siemers and Schnitzler 2004). Significant dietary overlap even when prey resources were limiting led Schoeman and Jacobs (2011) to conclude that competition does not structure the trophic niches of bat species, although they admit that greater differences in diet may be apparent when diets are quantified at finer resolution than at the ordinal level. Indeed finer resolution of dietary composition revealed the importance of the partitioning of minor prey items in reducing interspecific competition among coral reef fish, despite high overlap in the consumption of major prey items (Nagelkerken et al. 2009). Similarly, high dietary overlap in our study was primarily the result of the sharing of common prey items between the two cryptic bat species, while rare dietary items were generally consumed by a single species (Table 1).

We found that interspecific prey resource overlap was higher than intraspecific overlap, suggesting that individual *P. auritus* bats, in particular, are more likely to consume the same prey items as individuals from their sibling species, *P. austriacus*, than members of their own species. Greater levels of intraspecific dietary partitioning may be the result of higher intraspecific than interspecific prey resource competition because of differences in foraging habitat use. While *P. austriacus* preferentially forages in more open habitats, such as unimproved grasslands, or at the edge of riparian areas and woodlands (Razgour et al. 2011), *P. auritus* forages primarily in deciduous woodland (Entwistle et al. 1996). In this case, apparent resource partitioning may be less of an effect of competition for limited food resources than a pattern that results from habitat partitioning. The majority of grassland-only prey species, such as the moths *Agriphila tristella*, *Chrysoteuchia culmella*, and the crane fly *Tholera decimalis*, were consumed by *P. austriacus*, while woodland-only moth species, including *A. epomidion* and *Acronicta alni*, were consumed by *P. auritus*. Generalist prey species, such as *N. pronuba*, the most common prey item in the diet of both bat species, tended to be consumed equally by both species, hence resulting in the observed high dietary overlap. Other cryptic *Myotis* and *Pipistrellus* bat species show similar patterns of foraging habitat segregation mirrored by prey habitat associations (Arlettaz 1999; Davidson–Watts et al. 2006), though in both cases dietary partitioning was significant despite the coarse study resolution (Arlettaz et al. 1997; Barlow 1997).

The diet of the two cryptic bat species tended to only overlapped in summer, when the availability of Lepidoptera, the preferred prey resource, peaks in southern England (Jones 1990), suggesting that dietary resource partitioning is greatest in autumn, when prey resources are more limiting. In addition, interspecific competition may be greater in autumn because *P. austriacus* tends to forage in woodlands to a greater extent when ambient temperatures are low (Razgour et al. 2011). Therefore, it appears that when prey resources are more limiting and habitat use overlap is higher, the role of dietary partitioning in facilitating coexistence becomes more important. However, a larger dataset is needed to confirm the trend of seasonal differences in dietary niche overlap identified in our study.

Our results suggest that instead of prey selection driving differential habitat use (as is the case in Saunders and Barclay 1992), sympatric *P. austriacus* and *P. auritus* simply consume the most energetically beneficial prey available to them in their respective foraging habitats. Hence, spatial partitioning, rather than dietary partitioning, is the principal mechanism of coexistence among these highly cryptic bat species. It is still unclear what morphological differences, if any, drive the differences in foraging and flight behavior, because the two bats show no significant differences in wing loading and aspect ratio (Sevcik 2003), the main wing parameters controlling manoeuvrability and ability to sustain prolonged flight in bats (Norberg and Rayner 1987). Indeed, it could be that morphological divergence is not necessary to drive differences in habitat use by these species.

## Conclusions

HTS provided species-level identification of dietary components, and therefore allowed a more comprehensive comparison of the diet of two sympatric cryptic bat species. We found little evidence of trophic resource partitioning. Instead, patterns of prey consumption corresponded to predator habitat use, highlighting the importance of habitat partitioning as the principal mechanism of coexistence. The use of MOTUs as surrogates for prey species enabled us to overcome the constraints of an incomplete reference dataset, and corresponded well with prey species identification. Using generic arthropod primers, we were able to PCR amplify and sequence short fragments of degraded DNA from bat feces with little contamination from nonprey DNA, but our prey species identification confidence was compromised. Therefore, efforts should be put into testing and developing an optimal amplicon length for molecular diet studies, which will provide maximum identification ability with minimum loss of degraded prey DNA. Our study provides significant support for the use of molecular diet analysis, and in particular HTS, to decipher potential mechanisms of resource partitioning.

## Supporting Information

**Table S1.** *Plecotus austriacus*.

**Table S2.** *Plecotus auritus*.
