# Supplementary material for: High-throughput sequencing offers insight into mechanisms of resource partitioning in cryptic bat species
Source: Ecol Evol. 2011 Dec;1(4):556–70. doi: 10.1002/ece3.49 (PMC3287336; doi:10.1002/ece3.49)

# Supplementary 1 – Faecal sample collection dates and locations

# *Table S1.1 - Plecotus austriacus*

| **Date** | **Season** | **Location** |
| --- | --- | --- |
| 23.4.09 | Spring | Devon - 50°3N 3°3W |
| 23.4.09 | Spring | Devon - 50°3N 3°3W |
| 10.7.09 | Summer | Devon - 50°3N 3°3W |
| 30.7.09 | Summer | Devon - 50°3N 3°3W |
| 20.8.09 | Summer | Devon - 50°3N 3°3W |
| 20.8.09 | Summer | Devon - 50°3N 3°3W |
| 4.9.09 | Autumn | Devon - 50°3N 3°3W |
| 6.5.10 | Spring | Isle of Wight - 50°3N 1°2W |
| 26.5.10 | Spring | Devon - 50°3N 3°3W |
| 26.5.10 | Spring | Devon - 50°3N 3°3W |
| 26.5.10 | Spring | Devon - 50°3N 3°3W |
| 26.5.10 | Spring | Devon - 50°3N 3°3W |
| 26.5.10 | Spring | Devon - 50°3N 3°3W |
| 29.6.10 | Summer | Devon - 50°3N 3°3W |
| 6.7.10 | Summer | Devon - 50°3N 3°3W |
| 23.7.10 | Summer | Isle of Wight - 50°3N 1°2W |
| 5.8.10 | Summer | Isle of Wight - 50°3N 1°2W |
| 14.8.10 | Summer | Isle of Wight - 50°3N 1°2W |
| 29.8.10 | Summer | Devon - 50°3N 3°3W |
| 29.8.10 | Summer | Devon - 50°3N 3°3W |
| 29.8.10 | Summer | Devon - 50°3N 3°3W |
| 31.8.10 | Summer | Isle of Wight - 50°3N 1°2W |
| 31.8.10 | Summer | Isle of Wight - 50°3N 1°2W |
| 10.9.10 | Autumn | Isle of Wight - 50°4N 1°1W |
| 11.9.10 | Autumn | Isle of Wight - 50°3N 1°2W |
| 12.9.10 | Autumn | Isle of Wight - 50°3N 1°2W |
| 29.9.10 | Autumn | Devon - 50°3N 3°3W |
| 29.9.10 | Autumn | Devon - 50°3N 3°3W |

# *Table S1.2 -* Plecotus auritus

| **Date** | **Season** | **Location** |
| --- | --- | --- |
| 15.6.10 | Summer | Somerset - 50°6N 2°4W |
| 15.6.10 | Summer | Somerset - 50°6N 2°4W |
| 12.6.10 | Summer | Devon - 50°2N 3°5W |
| 3.7.10 | Summer | Devon - 50°5N 3°3W |
| 30.8.10 | Summer | Isle of Wight - 50°4N 1°3W |
| 31.8.10 | Summer | Somerset - 51°2N 2°1W |
| 31.8.10 | Summer | Somerset - 51°2N 2°1W |
| 31.8.10 | Summer | Somerset - 51°2N 2°1W |
| 31.8.10 | Summer | Somerset - 51°2N 2°1W |
| 31.8.10 | Summer | Somerset - 51°2N 2°1W |
| 13.9.10 | Autumn | Devon - 50°5N 3°3W |
| 15.9.10 | Autumn | Isle of Wight - 50°4N 1°3W |
| 15.9.10 | Autumn | Isle of Wight - 50°4N 1°3W |
| 15.9.10 | Autumn | Isle of Wight - 50°4N 1°3W |
| 15.9.10 | Autumn | Isle of Wight - 50°4N 1°3W |
| 15.9.10 | Autumn | Isle of Wight - 50°4N 1°3W |
| 15.9.10 | Autumn | Isle of Wight - 50°4N 1°3W |
| 1.10.10 | Autumn | Devon - 50°3N 3°5W |
| 1.10.10 | Autumn | Devon - 50°3N 3°5W |
| 1.10.10 | Autumn | Devon - 50°3N 3°5W |
| 1.10.10 | Autumn | Devon - 50°3N 3°5W |
| 1.10.10 | Autumn | Devon - 50°4N 3°4W |
| 1.10.10 | Autumn | Devon - 50°4N 3°4W |
| 1.10.10 | Autumn | Devon - 50°4N 3°4W |

# Supplementary 2 – PCR reaction conditions and cycle program (adapted from Bohmann *et al*. 2011, *FLoS ONE* 6, e21441)

PCRs were performed in 25 µl reactions using the Amplitaq Gold enzyme system (Roche, Basel, Switzerland). Each reaction contained 1 µl DNA extraction from faeces, 1x PCR Gold Buffer, 2.5 mM MgCl_2_ solution, 200 nM each dNTP, 0.1 µl AmpliTaq Gold, and 400nm of each primer. We used a DNAEngine Peltier Thermal Cycler (Bio-Rad Laboratories, Hercules, CA) with the following cycle program: Initial denaturation at 95°C for 4 minutes, followed by 40 cycles of 95°C for 15 seconds, 52°C for 30 seconds and 72°C for 30 seconds, followed by a final extension at 72°C for 6 minutes and 4°C forever. 5 µl of the PCR products were visualised on 2% agarose gels stained with ethidium bromide. Positive PCR-products were purified using the MSB(R) Spin PCRapace (Invitek, Westberg, Germany).

Real time qPCR was performed using a LightCycler (R) 480 II (Roche), and the enzyme Amplitaq Gold (Roche), in 25 µl reactions. Each reaction contained 1 µl DNA, 1x buffer, 2.5 mM MgCl_2_, 100 nM each dNTP, 0.1 µl Amplitaq Gold, 400nM of each primer, and 1 µl SYBR Green/Rox mix (Invitrogen, Carlsbad, CA).

# Supplementary 3

Seasonal variation in the proportion of prey orders identified in the diet of (a) *Plecotus austriacus* (Spring: N=8, Summer: N=14, Autumn: N=6), and (b) *Plecotus auritus* (Summer: N=10, Autumn: N=14) in southern England based on the results of the molecular diet analysis. White bars represent spring (April-May), grey bars summer (June-August) and black bars autumn (Sep-Oct). No *P. auritus* faecal samples were collected in the spring.


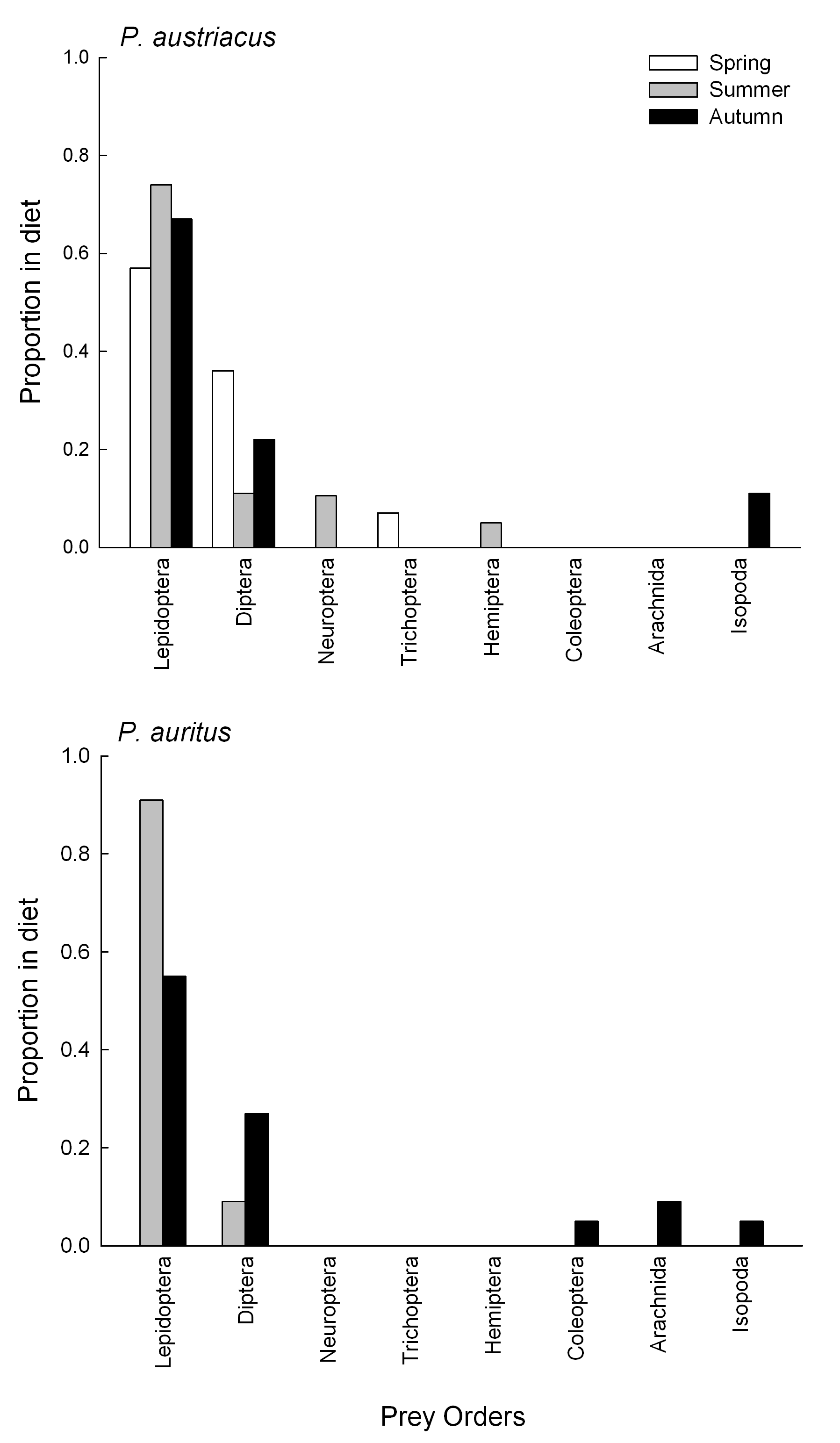

Supplement: Supplementary file 1 [file ece30001-0556-SD1.doc]
